# Synergistic Applications of Graphene-Based Materials and Deep Eutectic Solvents in Sustainable Sensing: A Comprehensive Review

**DOI:** 10.3390/s24082403

**Published:** 2024-04-09

**Authors:** Rossella Svigelj, Rosanna Toniolo, Cristina Bertoni, Alessandro Fraleoni-Morgera

**Affiliations:** 1 Department of Agrifood, Environmental and Animal Sciences, University of Udine, 33100 Udine, Italy; 2 Electrolux Italia SpA—Porcia, 33080 Pordenone, Italy; 3 Department of Engineering and Geology, University of Chieti—Pescara, 66100 Pescara, Italy

**Keywords:** graphene, deep eutectic solvents, sensors, ionic liquids, NADES, nanocomposites

## Abstract

The recently explored synergistic combination of graphene-based materials and deep eutectic solvents (DESs) is opening novel and effective avenues for developing sensing devices with optimized features. In more detail, remarkable potential in terms of simplicity, sustainability, and cost-effectiveness of this combination have been demonstrated for sensors, resulting in the creation of hybrid devices with enhanced signal-to-noise ratios, linearities, and selectivity. Therefore, this review aims to provide a comprehensive overview of the currently available scientific literature discussing investigations and applications of sensors that integrate graphene-based materials and deep eutectic solvents, with an outlook for the most promising developments of this approach.

## 1. Introduction

Sensing strategies are evolving towards technologies more and more focused on ultra-low detection thresholds and highly selective devices. These performances can be enabled by nanotechnologies, operated thanks to either lithographically defined, top-down structures [1,2,3] or chemically/biochemically obtained, bottom-up constructs [4,5,6]. A kind of bridge between the top-down and bottom-up approaches can be represented by graphene-based nanostructures. Graphene is a two-dimensional material composed of a single layer of carbon atoms arranged in a hexagonal lattice structure [7]. Andre Geim and Konstantin Novoselov isolated and characterized graphene in 2004, an achievement for which they were awarded the Nobel Prize in Physics in 2010 [8]. A scientific database study carried out in November 2023 using the keyword “graphene” yielded over 203,000 papers, including approximately 10,000 review papers. The peculiar physical properties of the material, which have been described in detail in uncountable excellent reviews (see, for example, [9,10,11]) allow for its use in almost infinite applications, covering different technologically and scientifically relevant fields of today’s human activity. Among some of the most successful and/or investigated ones, it is possible to mention general electronics and optoelectronics, for which the presence of graphene and its derivatives improves the electronic transport of the devices [12,13,14,15]; energy-related applications [16,17], in which, again, the electronic transport ability of graphene helps to improve the overall properties of, for example, batteries and capacitors; catalysis [18,19], a field that exploits both the ultra-high surface area achieved by graphene/graphene derivatives and their enhanced electron transport properties for increasing the overall yields of chemical reactions; medicine [20,21,22,23], in which the ability of graphene derivatives (in particular of graphene oxides) to interact with biomolecules is used to implement drug delivery, to provide selective electromagnetic absorption for thermally destroying cancerous cells, for imaging, and for many other biomedical purposes [24,25]; the mechanical reinforcement of composites and/or the functional modification of composites, fields in which the exceptional mechanical resistance of graphene derivatives is often exploited by creating materials able to withstand very demanding mechanical stresses [26,27,28,29,30]; and, of course, sensing, for which both the ability to provide very large surface areas for enhanced interactions with analytes and the intrinsic high-charge-transport properties of graphene/graphene derivatives lead to notable performances of the realized devices [31,32,33,34].

In fact, in recent studies, graphene and its derivatives have emerged as highly effective materials for detecting analytes, demonstrating remarkable capabilities in both the liquid [35,36] and gas [37,38,39] phases. In some cases, graphene-based species were able to provide limits of detection for liquid-phase sensors as low as 52 pg/L for selected molecules [40], while, in the gas phase, limits of detection down to a few molecules have been reported [41]. Regarding gas sensing, high sensitivity to various gases, including ammonia, nitrogen dioxide, and methane, has been demonstrated [42,43,44]. Moreover, graphene has evidenced a huge potential for biosensing, demonstrating the ability to detect biomolecules, such as DNA, proteins, or enzymes, with high sensitivity [45]. Indeed, a graphene-based device was able to detect a single bacterium in 1 μL of liquid [46]. In all the best-performing devices, graphene or graphene derivatives have been functionalized with various chemical moieties in order to enhance both the selectivity and sensitivity, and a variety of surface modifiers have been used: acids, bases, alcohols, DNA, proteins, oligomers, enzymes, and many others [47,48,49].

Attempts to modify graphene and its derivatives with ionic liquids (ILs) have been extensively made in the past. ILs are organic salts with low melting points (usually below 100 °C), obtained by the combination of an organic cation, commonly imidazolium-based cations, with a diverse array of anions, such as Cl^−^, BF_4_^−^, PF_6_, and NTf_2_ [50]. This feature allows them to be used in a variety of applications requiring high electrical ionic conductivity and the liquid state, like, for example, lithium-based batteries [51]. However, ILs are expensive and may pose environmental pollution problems due to their high stability and affinity for lipidic membranes, and viable alternatives for these compounds are actively sought [52]. For these reasons, in recent years, DESs have emerged as a more economical and environmentally friendly potential substitute for ILs [53,54].

**Figure 1:**
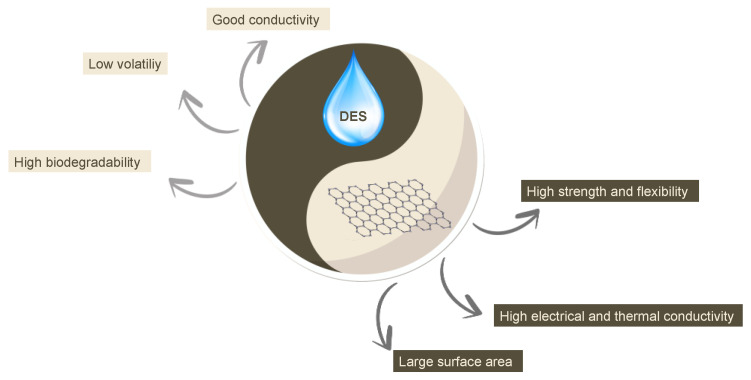
Overview of the ideal coupling of the general characteristics of DESs and graphene-based materials for sensing applications.

DESs have the major advantages of ILs (good electrical conductivity, low volatility) with a high biodegradability due to their intrinsic chemical structure, and hence, they are very interesting compounds to couple with graphene-based materials for designing novel devices with high performances and environmental sustainability (Figure 1).

**Figure 2:**
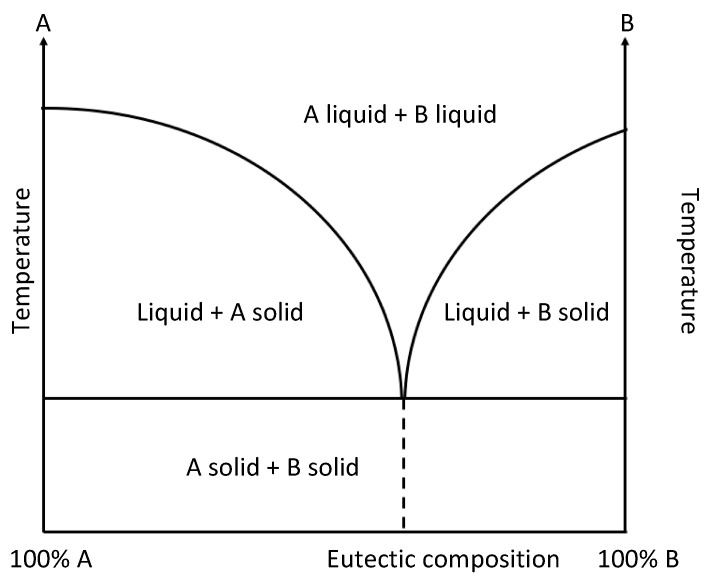
Eutectic-phase diagram.

DESs are obtained by simply mixing, in appropriate stoichiometric ratios, two components: a hydrogen bond acceptor (HBA) and a hydrogen bond donor (HBD) [55]. HBAs are usually high-melting-point salts, such as halides of tetra-alkylammonium, phosphonium, and quaternary ammonium, while HBDs are generally chosen among the classes of alcohols, carbohydrates, amines, or amides [56]. The mixing in specific stoichiometric ratios of the pure starting compounds, followed by heating, under stirring at temperatures between 50 and 100 °C for 2–4 h and at atmospheric pressure allows for the obtainment of a homogeneous eutectic liquid mixture, as shown in Figure 2 [57]. No additional solvent or traditional reactions are necessary, eliminating the need for purification procedures. This simplicity enhances their potential as cost-effective alternatives to traditional organic solvents and ionic liquids (ILs).

DESs are highly viscous and dense liquids with low volatility, high thermal and electrochemical stability, low flammability, good conductivity, and the ability to solvate both organic and inorganic compounds [54,58]. Their significant biodegradability and remarkably low toxicity levels, together with their aforementioned properties, allow for their full qualification as “green solvents” [59,60]. Moreover, the starting reagents for their production are easily available, which makes them even more attractive compared to classic organic solvents and ILs.

The density of DESs, due to their molecular organization, is usually greater than that of the starting components. This is due to the fact that when the HBDs and HBAs are mixed, the spaces between the molecules decrease, leading to an increase in the density value (the so-called “hole theory”) [61]. Their viscosity is usually high, due to both intermolecular forces, regulated by hydrogen bonds (the starting constituents of the DESs include a high number of hydroxylic groups), van der Waals and electrostatic interactions, and the large size of the ions, as well as the reduced free volume that characterizes the medium [57]. The polarity varies according to the considered type of DES, with the HBA/HBD molar ratio having a major influence on this parameter. For example, in the specific case of a DES composed of choline chloride (ChCl) and glycerol, an increase in the concentration of ChCl (HBA) corresponds to a linear increase in the polarity. Taken all together and coupled with their high conductivity, these properties make DESs very interesting systems for electrochemical applications, as they can perform the role of both solvent and electrolyte at the same time [62].

Finally, they can be obtained from fully natural compounds, like amino acids, sugars, and choline and organic acids, creating a subset of even more environmentally friendly DESs called “Natural Deep Eutectic Solvents” (NADESs) [63], with intermediate properties between those of an aqueous medium and a lipid medium [64]. Indeed, NADESs can solubilize poorly soluble metabolites in water and contribute to the synthesis of intracellular macromolecules [65]. The presence of these compounds in plants and animals, for example, favors the survival of the considered species, even in conditions of low temperatures or small quantities of water [66].

DESs have found application in many different fields [67], like chemical analysis, as extracting solvents [68], in chromatographic and electrophoretic separations [69], and in the fabrication of electrochemical sensors [70,71,72,73]. Their use leads to more effective and selective extractions, thanks to the possibility of modulating their hydrophobic/hydrophilic characteristics. DESs have also been used to extract metals [74]; bioactive compounds, such as flavonoids [75,76] and phenols and polyphenols [77]; saponins, anthraquinones, and other molecules from natural matrices [78,79]; and biopolymers, such as lignin [80], starch [81], and proteins, such as gluten, from food matrices [82]. In terms of separative techniques, DESs have been used as mobile-phase modifiers in liquid chromatography [83]. Finally, in the electroanalytical field, they have been used simultaneously as electrolytes and preconcentrating solvents in the development of gas sensors [84,85] and biosensors aimed at determining gluten [86,87].

The integration of graphene with deep eutectic solvents (DESs) presents several benefits: firstly, it enhances the conductivity of the composite materials, opening avenues for the creation of cutting-edge, highly sensitive sensors; additionally, they play a crucial role in preventing the degradation of the graphene layers, thereby ensuring the longevity and durability of the material; lastly, by carefully selecting appropriate DESs, it becomes feasible to dissolve analytes that are typically insoluble, broadening the scope of potential applications. These intriguing advancements are further exemplified by a growing interest in the technological field, as evidenced by recent patent filings targeting the production of graphene through the innovative application of DESs [88,89].

Because the approach of coupling graphene and DESs is still young, to the best of our knowledge, this review represents the first comprehensive examination of the combined use of these two materials for sensor development; furthermore, this overview is complemented by a few selected cases of the use of ILs instead of DESs, in order to highlight the ongoing transition between ILs and DESs in their use coupled with graphene-based materials.

In most cases, DESs have been used as solvents for the extraction/preconcentration of the analyte; only in a few works have DESs been reported as functionalizing groups of the graphene derivative to enhance the sensing performance of the resulting device. An outlook of the possible developments completes the description of this young but promising field.

## 2. Enhancing Sensor Performance through DES–Graphene Integration

As mentioned in the Introduction, before describing specific work on graphene/graphene derivatives and DESs, it is useful to describe some previous work on using ILs.

**Figure 3:**
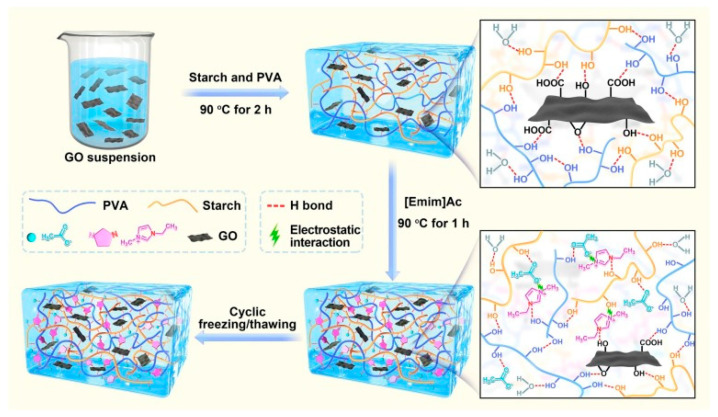
Illustration of the design strategy and formation mechanism of the starch-based hydrogel described in [90]. Reproduced with permission from Elsevier.

For example, Cui et al. paired graphene oxide with ILs to create a versatile hydrogel employing a new physical-crosslinking method [90]. In their work, polyvinyl alcohol was selected to create a dual network alongside starch molecules, utilizing its biocompatibility, biodegradability, and mechanical strength, while the ionic liquid enhanced the chemical network compatibility and further increased its mechanical properties. The incorporated graphene oxide (GO) contains a high number of hydroxyl, carboxyl, and epoxy groups, which help to stabilize the network (Figure 3). The authors postulated that the combined integration of IL and GO would boost the mechanical strength, conductivity, and resistance to freezing of the starch-based hydrogel, aiming to craft flexible, versatile, wearable sensors. This innovative material was employed in the fabrication of wearable sensors, capable of detecting tensile stress, compression, and temperature (with a sensitivity of 0.71%/°C). Furthermore, the researchers showed that the device had excellent stretchability (657.5%) and strength (0.64 MPa), high conductivity (1.98 S⋅m^−1^), and good anti-freezing ability (still working even at −20 °C). These sensors were then employed to monitor human motion, pressure, and body temperature.

Li et al. utilized graphene quantum dots and an IL to create an electrode for the detection of rutin [91]. With this set-up, they noticed an enhancement of the differential pulse voltammetry signal, as well as of both the effective surface area and the conductivity of the sensor, resulting from the synergy between the graphene quantum dots and the IL. These features allowed them to develop a sensor capable of detecting rutin with a linear response within the concentration range from 5 × 10^−9^ to 1 × 10^−5^ mol L^−1^ and a limit of detection (LOD) of 2 × 10^−9^ mol L^−1^.

Shabani-Nooshabadi et al. utilized CuO/reduced graphene nanoribbon nanocomposites and an IL (1-ethyl 3-methyl imidazolinium chloride) to simultaneously detect the biologically active molecules tramadol, olanzapine, and acetaminophen [92]. The incorporation of the IL-incorporating nanocomposite in this novel sensor resulted in an increased surface area, outstanding conductivity, and a superior electrocatalytic performance. The sensor demonstrated a linear detection range of 0.08–900 μM and a low detection limit of 0.05 μM specifically for tramadol.

Zhao and colleagues exploited graphene combined with ILs in a sensor for the determination in the liquid phase of sunset yellow, a synthetic dye used in the food industry [93]. The authors used a glassy carbon electrode coated with an IL-functionalized graphene and a molecularly imprinted polymer (MIP) suspension. They took advantage of the fact that some IL components can interact with hydrophilic template molecules, preparing IL-based water-compatible MIPs and, in this way, increasing the ability of the MIPs to recognize hydrophilic analytes. However, MIPs are not always conductive; hence, the researchers introduced graphene to improve this aspect. Furthermore, the combination of ILs and graphene led to a more stable composite, with a better electrochemical performance, reaching an LOD of 4 nM.

The above-mentioned results obtained with the graphene/graphene derivatives and ILs are clearly promising, but the researchers are aware of the high costs and environmental concerns for this latter class of compounds; therefore, in recent years, the concept of coupling ionic compounds and graphene derivatives has been gradually shifting towards more environmentally friendly ionic materials, like DESs. The first example appeared in 2015 by Hayyan et al., who prepared 18 ammonium- and phosphonium-based DESs and employed them for the subsequent modification of GO with KMnO_4_ via a simple reaction carried out at 60 °C for 3 h under sonication [94]. Thanks to the DESs, they obtained different levels of GO reduction and, in some cases, both functionalization and reduction were achieved. The FTIR analyses showed that their DES 5 (Choline Chloride/Urea, 1:2) was the most affecting agent among the DESs, and five new peaks were detected after treatment, all of which corresponded to peaks in the DES spectrum. The bands between 3326 and 3186 cm^−1^ represented –NH_2_ and –NH stretching vibrations, whereas the in-plane stretching of –NH was evident at ~1605 cm^−1^. These observations indicate that the treatment of graphene with KMnO_4_ (pH = 0.14) and DES 5 led to changes in the functional groups obtained from the DES on the carbon surface. New peaks were also observed in the XRD patterns, suggesting alterations in the crystalline structure of the graphene. This indicates that the treatment with the DESs led to changes in the arrangement of the atoms in the graphene lattice, possibly due to the introduction of functional groups or other chemical modifications. However, not all the DESs produced new functional groups. The main result obtained after characterizing the material with IR spectra, TGA/DTG, XRD, SEM, and TEM was that a change occurred in the surface chemistry of the material, which resulted in a few DES-functionalized graphene oxides (namely, those functionalized with choline chloride/urea, N,N-Diethylethanolammonium chloride/ethylene glycol, N,N-Diethylethanolammonium chloride/triethylene glycol) showing an improved dispersion and stability in water with respect to the simple oxidized graphene. The importance of this paper lies primarily in the first demonstration of the easy possibility of achieving DES-functionalized graphene species, using a fast and inexpensive method (previous oxidation of the graphene moiety and the use of an appropriate DES).

Another approach, based on the fabrication of a novel reduced graphene oxide-supported nickel cobaltate nanorod composite (RGO-NiCo_2_O_4_) was proposed by Shao et al., who used this material for realizing a nonenzymatic electrochemical sensor for glucose [95]. In more detail, the researchers used a choline chloride/urea DES as a solvent for achieving, at relatively low temperatures (110 °C) and within a reasonable amount of time (about 20 h of total treatment), NiCo_2_O_4_ nanorods grown on reduced graphene oxide (RGO) used as the active electrode, obtaining, after an annealing step conducted at 300 °C for 3 h, a very large electrocatalytic active area on the RGO surface. The excellent electrical conductivity of the RGO-based electrode and its high active surface allowed for the achievement of good electrocatalytic activity toward glucose oxidation in the alkaline solution: the sensor showed a wide linear range between 1 μM and 25 mM, and a detection limit towards glucose of 0.35 μM.

In another study, Hu and co-workers synthesized an electrochemically reduced graphene oxide (ERG) on a glassy carbon electrode (ERG/GCE) [96]. Subsequently, a polythionine–methylene blue (PTH-MB) polymer was electropolymerized on the electrode surface in a phosphate-buffered saline (PBS) solution with a pH of 6. The electropolymerization was carried out incorporating a 50% (*v*/*v*%) DES solution containing thionine and methylene blue, confirming previous reports that have suggested that the use of DESs as solvents in electropolymerization is beneficial for obtaining high surface areas and better electrochemical activities of the obtained polymers [97]. These modified electrodes were then evaluated for their performance in the direct electrocatalytic oxidation of NADH, a crucial coenzyme associated with various physiological processes, such as cell proliferation, tumor formation, ischemia, and certain brain diseases. The sensor exhibited a good linear range spanning from 1.52 μM to 3333.33 μM, accompanied by a notably low LOD of 0.51 nM.

**Figure 4:**
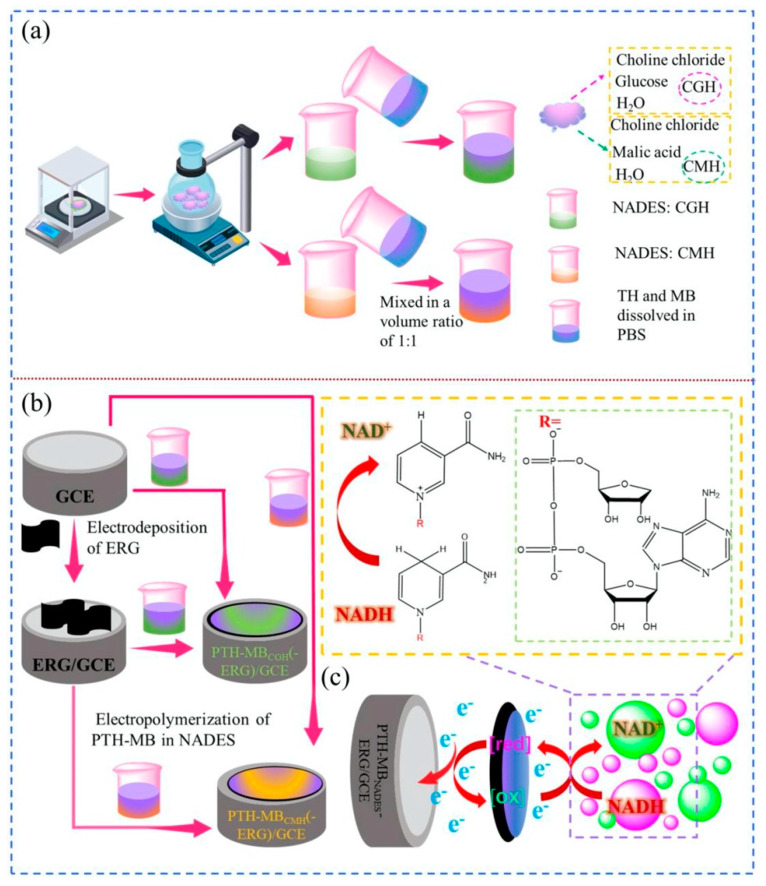
(**a**) Schematic procedure for preparation of NADESs and electropolymerization solutions. (**b**) Fabrication of the PTH-MB_NADES_ and PTH-MB_NADES_-ERG electrodes. (**c**) The proposed NADH measurement mechanism on the proposed electrode [98]. Reproduced with permission from Elsevier.

Again, Hu and collaborators proposed a modification of a glassy carbon electrode for the electrocatalytic oxidation of NADH with an approach similar to the previously mentioned one, except for the fact that they used a ternary NADES (choline chloride, malic acid, and H_2_O at a molar ratio of 1:1:2, and choline chloride, glucose, and H_2_O at a molar ratio of 1:1:11) as the DES (see Figure 4) [98]. Excellent electrocatalytic activity was displayed by the sensor functionalized with this combination of materials, with a high degree of reproducibility (the maximum relative standard deviation (RSD%) ranged from 3.65 to 4.32%), a large linear range (0.51–3333.33 µM), a low LOD (0.159 nM), and good stability (4 weeks). Moreover, the sensing composite material is simple to prepare and responds quickly to NADH (response time: 3 s). The resulting sensor was used to test urine samples, revealing good baseline recovery rates (between 89 and 102% of the original baseline). Using the same electrochemical reduction in GO and the electropolymerization of thionine–methylene blue in the NADES electrolyte solution described in the previously discussed Ref. [96], the authors effectively proved a straightforward, quick, environmentally friendly technique for the synthesis of nanocomposites. A particular point of interest in this work is that, without the use of additional specific reagents and enzymes, the oxidation of NADH occurs at a low potential, preventing the poisoning of the electrode surface.

In another interesting study, Gollas et al. explored the electrochemical behavior of graphene in the DES choline chloride/ethylene glycol (12CE), and the potential of this couple for electrochemical applications [99]. In more detail, the study measured the graphene potential window in the 12CE and estimated the apparent electron transfer kinetics of an outer-sphere redox couple. The 12CE electrolyte was also employed to fabricate nanostructured metal (Zn) and metalloid (Ge) hybrids with graphene by electrodeposition. The findings reveal the significant impact of graphene’s two-dimensional structure on DES electrochemistry, resulting in a spatially varied zinc deposition and graphene degradation during potentiodynamic Zn deposition. The cathodic regime’s reduced stability was attributed to the electrochemical generation of radicals during choline reduction, leading to the degradation of the monolayer graphene. However, the degradation and spatial inconsistencies in the deposits could be mitigated by potentiostatic deposition at lower cathodic potentials, as evidenced by the uniform electrodeposition of germanium. These discoveries hold importance for the processing of graphene and related carbon materials in choline chloride-based DESs and their utilization in such electrolytes.

Kumar et al. evaluated the behavior of rhodamine B (RB) in the DES called “Reline” (choline chloride–urea) with or without GO [100]. The researchers explored the synergistic effects of the GO and DES on the photophysical processes by changing the solvent nature and GO amounts, finding that the fluorescence of RB can be notably altered (either enhanced or quenched) by the presence of a surfactant or GO. The intensity of the fluorescence is influenced by the RB concentration at the surfaces of the GO or in the Reline layer. The results of this investigation suggest that the basic nature of the Reline DES may produce an RB zwitterion, which can form a DES−monomer ion pair, leading to sizeable fluorescence intensity. The aforementioned behavior holds promise for potential applications in chemical sensors or biotechnology exploiting optical detection means.

**Figure 5:**
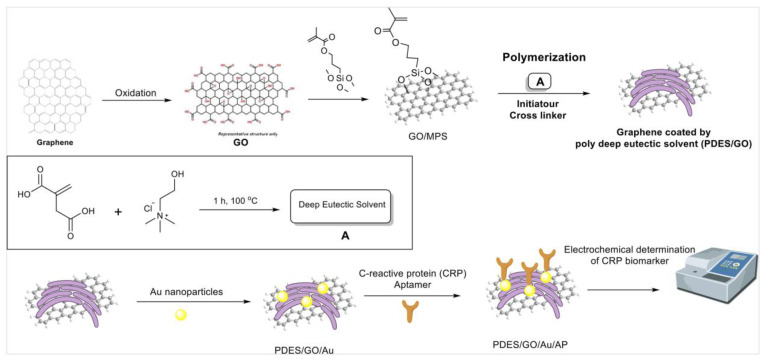
Schematic fabrication process of the C-reactive protein aptasensor based on graphene oxide /PolyDES/Au NPs. Reproduced with permission from Elsevier [101].

In a recent work, Mahyari et al. proposed an aptasensor (i.e., a sensor based on aptamers) based on GO and a gold nanoparticle nanocomposite modified with a deep eutectic solvent for the detection of C-reactive protein biomarkers (Figure 5) [101]. The resulting nanocomposite was highly dispersible and stable in the chosen medium, with a high level of porosity in the range of about 20–25 nm, granting a superior functionality and surface charge density for the device. In particular, the sensor demonstrated high sensitivity (LOD = 0.0003 ng mL^−1^), selectivity (tested against interfering agents such as alpha-fetoprotein, lysine, and uric acid, for which the sensor showed a negligible response), reproducibility (the RSD was found to be 4.6%), and stability (the sensor proved to be stable for 10 days), and with a linear range of 0.001–50 ng mL^−1^. In this strategy, the nanocomposite was used as a carrier as well as a signal enhancer; moreover, it is clearly also possible to use this approach for other analytes by changing the selected aptamer. 

**Figure 6:**
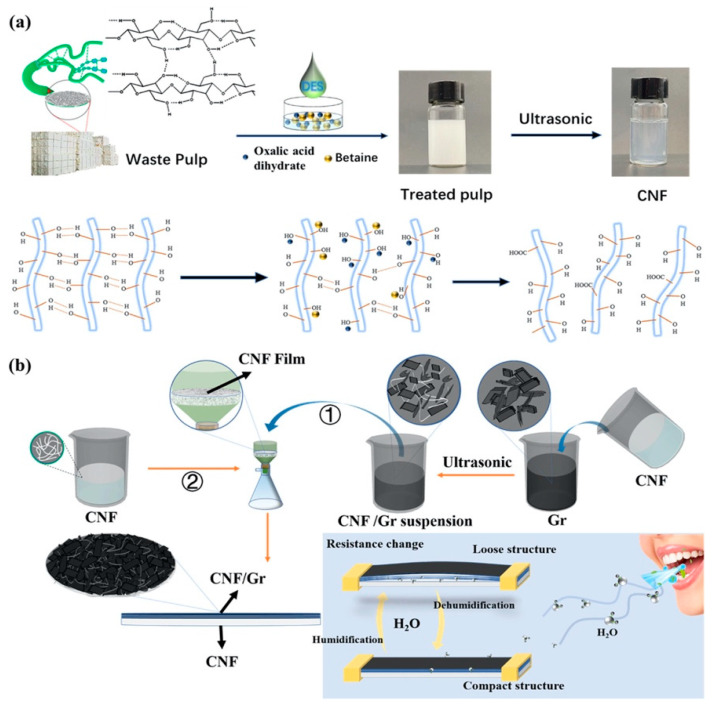
(**a**) Scheme of the extraction process of CNFs from waste pulp. (**b**) Schematic diagram of the preparation of a humidity sensor by employing CNF-dispersed graphene (①) as the humidity-sensing layer. The latter was filtered onto the coessential CNF film surface to form the sensor (②). Reprinted (adapted) with permission from the American Chemical Society [102].

Recently, Yao et al. proposed the construction of a sensor based on cellulose nanofiber (CNF)-dispersed graphene (Gr) as a humidity-sensing layer (Figure 6) [102]. In this sensor, a DES (oxalic acid/betaine) was used as the solvent for the extraction of the CNFs from waste pulp. Graphene powders (10, 20, and 40 mg) were introduced into a solution of CNF with a concentration of 0.1 wt%, followed by ultrasonic treatment for 3 h. This procedure led to the creation of a uniformly dispersed suspension of CNF/Gr. Subsequently, the suspension was effortlessly filtered onto a CNF film to construct the humidity sensor’s sensing element. Because of their great surface areas, the CNFs offered adsorption sites for capturing water molecules and facilitated the electron transfer from the adsorbed water molecules to the graphene. This process further heightened the electrical signals when the sensing layer underwent hygroscopic mechanical expansion in humid conditions. The sensor provided a satisfactory response time (45 s) and recovery time (33 s), low hysteresis (4%), a wide RH detection range (15–99%), and long-term stability (15 days). Moreover, it had the ability to monitor the humidity levels of human skin and breath, showcasing a flexible, non-contact humidity-sensing capability achieved by adjusting its CNF-to-Gr component ratio as needed.

**Figure 7:**
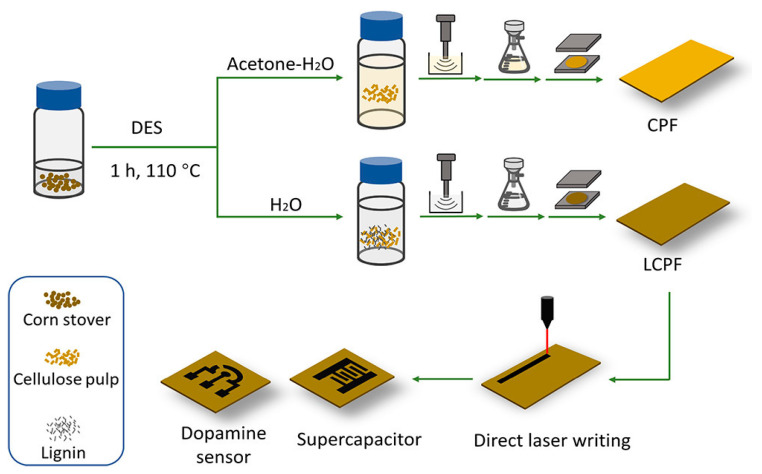
A schematic illustration depicting the fabrication process of biomass-based sensors employing DESs is presented. Initially, corn stover undergoes pretreatment with a DES, followed by the addition of an antisolvent (such as an acetone–water mixture or water). Subsequently, the pretreated slurry is ultrasonicated and vacuum-filtrated to produce wet films. These wet films are then subjected to hot pressing for the creation of laser-induced graphene (LIG), which is further utilized for the production of on-chip supercapacitors and dopamine sensors through direct laser writing. Reprinted with permission from the American Chemical Society [103].

A recent study by Wan et al. explored the conversion of lignocellulosic biomass into porous graphene using direct laser writing (DLW) and DESs, including choline chloride:oxalic acid, choline chloride:formic acid, and choline chloride:ethylene glycol (Figure 7) [103]. This process has been termed *laser-induced graphene* (LIG). The study found that the cellulose pulp resulting from pretreatment with a choline chloride oxalic acid DES was a suitable substrate for LIG formation, and the obtained LIG exhibited a 3D porous structure and high crystallinity. It was suggested that pseudo-lignin from the DES-treated cellulose pulp helped to produce the LIG. The LIG-embedded films showed good electrochemical characteristics when utilized to create on-chip supercapacitors and dopamine sensors, with a linear range between 1 × 10^−6^ and 40 × 10^−6^ M and an LOD of 0.659 × 10^−6^ M. Thus, it was further demonstrated that DESs can act as enabling auxiliary compounds for the production of lignocellulose-derived compounds, which can be transformed into porous graphene materials in large quantities, and that these materials could have a wide range of uses for effective, inexpensive, and even disposable electronics.

**Figure 8:**
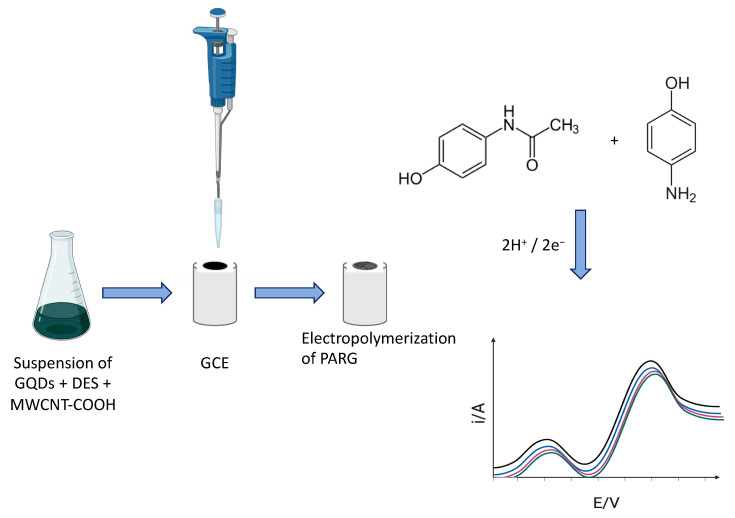
Schematic illustration of the preparation of electrodes for the detection in the liquid phase of paracetamol and 4-aminophenol, electrode modification was followed by DPV measurements. Adapted with permission from the RSC [104].

In yet another work, Fotouhi et al. proposed the development of an electrochemical sensor designed for the precise analysis of paracetamol and 4-aminophenol with enhanced sensitivity [104]. The sensor was fabricated by electropolymerizing L-arginine onto the surface of a glassy carbon electrode modified with a nanocomposite consisting of graphene quantum dots, a DES (choline chloride–urea, 1:2), and carboxyl-functionalized multiwall carbon nanotubes, as shown in Figure 8. Hence, in this sensor, the DES played an active role as part of the composite sensing layer. The sensor exhibited an excellent performance for the analytical monitoring of paracetamol and 4-aminophenol, with wide linear dynamic ranges (from 0.030 to 110 mmol L^−1^ and 0.050 to 100 mmol L^−1^) and LODs of 0.010 mmol L^−1^ and 0.017 mmol L^−1^, respectively. The practical applicability of the sensor was explored by the determination of both compounds in human fluid samples, with recoveries between 97 and 102% of the baseline. The article also discusses the advantages of electrochemical methods and polymer-modified electrodes, as well as the properties and applications of graphene quantum dots and deep eutectic solvents.

More recently, a new method to immobilize MIPs on the surface of reduced graphene oxide (rGO) through covalent bonding has been proposed by Cao and colleagues [105]. MIPs are frequently used in electrochemical sensing, as they can be engineered to selectively detect a solute analyte. However, they are limited in terms of their electrochemical activity, conductivity, and absorption capacity (affecting their sensitivity); hence, they are usually coupled to conductive nanoporous materials, like, for example, graphene or graphene derivatives. In this work, DESs were explored as solvents for the rGO surface modification prior to the covalent functionalization of the latter. In particular, the surface of the rGO was modified with maleic anhydride via a Diels−Alder reaction, employing a DES-based solution prepared using ZnCl_2_ and choline chloride. Next, 3-propyl-1-vinylimidazolium molecular units were anchored and polymerized in the presence of ethylene glycol dimethacrylate (EGDMA) using chloramphenicol (CAP) as the template. The impact of the varying molar ratios of the individual precursors on the adsorption capacity of the synthesized materials was examined, culminating in the fabrication of an electrochemical sensor for detecting CAP. The covalent bonding of the MIP units enhanced the sensitivity of the sensor, with an LOD of 0.204 μM and a linear range between 0.2 and 4.0 μM. The authors opted to utilize p-nitrophenol and thiamphenicol as interfering molecules to evaluate the specificity of MIP-rGO towards CAP. They obtained a heightened sensitivity and specificity by achieving relatively low current intensities in the presence of the interfering molecules.

With regard to NADESs, Silva et al. proposed an electrochemical sensor for the detection of oleuropein (OLE) based on the use of this class of solvents coupled to graphene [106]. The authors suggested that the addition of a NADES to the supporting electrolyte provided better results in the electrochemical detection of phenolic compounds. This approach was coupled to the use of graphene and multiwalled carbon nanotubes (MWCNTs), which are known to enhance the sensitivity of the voltammetric response. Regarding the supporting electrolyte, in this work, the best OLE voltametric peaks were obtained with a 5 mM Britton–Robinson buffer (BRB) at a pH of 9. Three different NADESs were then synthetized and added to the selected supporting electrolyte, and the best electrochemical signal was obtained with the NADES containing 10% (*v*/*v*) of lactic acid, glucose, and H_2_O (LGH). The methodology involved the use of a disposable pencil graphite electrode (PGE) as the working electrode, modified by dipping it in graphene oxide (GO) and MWCNT dispersions in water. The GO-modified PGE electrode (GOPGE) provided oxidation peak currents for OLE that were 1.3 times higher than those of the bare PGE. Among all the combinations tested, the authors state that the LGH-GOPGE system resulted in a signal enhancement 5.3 times higher than that of the bare electrode with the unmodified buffer. Additionally, the peak potentials recorded for the LGH-GOPGE system exhibited a mild shift to positive oxidation values in comparison to the peak currents recorded for the OLE in the unmodified buffer. The positive shift was attributed to the strong interaction of the OLE with the NADES H-bond. The electrochemical behavior of the OLE was then evaluated using differential pulse voltammetry. The proposed electrochemical sensor was successfully applied to the determination of OLE in an olive leaf extract prepared by ultrasound-assisted extraction, with a satisfactory linear range between 0.10 and 37 μM and an LOD of 30 nM.

To complete this review of the use of coupled graphene/graphene derivatives and DESs in sensors, Table 1 summarizes the most significant aspects of the discussed devices, including their most prominent characteristics and peculiar aspects, as well as the reported (when available) features of their performances reproducibilities/repeatabilities/stabilities.

## 3. Conclusions and Outlook

The integration of graphene-based materials and DESs aimed at sensing is a relatively new field that began to be explored in the mid-2010s. Despite currently being in its early stages, these sensors have demonstrated noticeable performances, such as remarkable linearity ranges (spanning over three orders of magnitude), low limits of detection (in the tens-of-nanomolars range), and satisfactory selectivities.

Throughout this review, we have reported how DESs can be effectively utilized for the modification of graphene, resulting in different levels of reduction and functionalization. First of all, the realization of DES-functionalized graphene is straightforward and accessible and can be easily followed by simple FTIR spectrometry, as these modifications lead to detectable changes in the surface chemistry of the latter. DESs have also been used to produce actual composites, which can be used as the sensing layers for different analytes, from glucose to paracetamol.

This approach has several points of interest:

- It allows for the coupling of well-known high-performing semiconductor/conductors, such as graphene-related moieties, with chemically versatile solvents, like DESs. This opens numerous opportunities for the development of selective and high-performing sensors and biosensors;
- DESs are truly eco-friendly, and they are really inexpensive (choline chloride, one of the most used building blocks for DESs, is produced in the range of about 150–170,000 tons/year);
- The combination between the two materials allows for an unprecedented combination of advanced electronic and chemical properties at an extremely low cost, with a negligible environmental impact.

In this view, the aforementioned combination of graphene derivatives and DESs can ensure a decisive step ahead for precise and effective sensing. To fulfill this vision, however, there are still open problems that must be addressed and solved. Firstly, a significant challenge is related to the high viscosity of DESs. Their intrinsic chemical structure (with plenty of ionic groups and hydroxylic group chains) results in very high viscosities, which limit mass diffusion (hence, the speed of the response and, in some cases, the cyclability of the devices also create hurdles to reaching low limits of detection). This issue can be partially solved by limiting the concentration of DESs in the graphene-derived material/DES mixture, or by using the composite at higher temperatures.

Secondly, the limited solubility of graphene and its derivatives in DESs poses another unresolved challenge. This limitation might restrict the presence of the electronic/ionic conductor in the heterogeneous sensing layer, potentially diminishing the overall sensor performance. 

Lastly, the shelf life of mixed DES/graphene-derived materials could be limited due to issues related to phase separation and the degradation of the DESs. Most of these current practical challenges linked to DES/graphene-based materials can be resolved relatively easily through the rational design of chemical compounds. By employing smart design strategies for sensing devices, such as highly nanostructured sensing surfaces, porous layers, and flow devices, it is possible to effectively tackle challenges and achieve a superior performance in liquid-phase and gas-phase analyses. Integrating these approaches with lab-on-a-chip and microfluidic technologies could ideally bridge the existing gaps. Scientists are becoming increasingly interested in this novel field, and new discoveries are expected to influence sensing strategies, particularly in the food and biomedical industries, within a few years.

## Figures and Tables

**Table 1:** Graphene–DES composites used in sensor development and their performances. Sensor devices along with their respective characteristics are presented. For brevity, we focus on detailing materials that significantly enhanced the sensor performance.

Target Analyte	Type of DES	Type of DES-Based Graphene System	Role and Function of the DES–Graphene Composite System	Sensing Method	Linear Range	LOD	Stability–Reproducibility–Repeatability	Reference
Glucose	ChCl–urea mixture (CU-DESs)	Reduced graphene oxide-supported nickel cobaltate nanorod composite (rGO-NiCo_2_O_4_ nanorods)	Electrocatalytic activity toward glucose oxidation in alkaline media; enhanced electrical conductivity.	Amperometry (nonenzymatic)	1 μM–25 mM	0.35 μM	Stability: sensor current response to 1 mM glucose in NaOH solution was stable above 90% of the initial response up to 1800 s. Reproducibility: anodic peak currents of four independently prepared RGO-NiCo_2_O_4_/Nafion/GCE electrodes showed 1.92% relative standard deviation (RSD). Repeatability: ten anodic peak current measurements on the modified electrode showed RSD of 1.98%.	[95]
Nicotinamide adenine dinucleotide (NADH)	Choline chloride:ethylene glycol (ChCl-EG, CE)	Polythionine–methylene blue (PTH-MB) electropolymerized in deep eutectic solvent (CE)–electrochemically reduced graphene oxide (ERG)-modified GCE glassy carbon electrode (PTH-MB_CE_-ERG/GCE)	Electropolymerization of PTH-MB film in CE provides improved stability and sensitivity and a significant reduction in LOD value; ERG film facilitates electron transfer.	Cyclic voltammetry (CV)	1.52 μM–3.33 mM	0.51 nM	Stability: the sensor response to 1.0 mM NADH showed a loss in sensitivity by ca. 12% after 28 days.	[96]
Nicotinamide adenine dinucleotide (NADH)	Natural DES (NADES)	Composite electrode based on electrochemically reduced graphene oxide (ERG)/poly(thionine–methylene blue) (PTH-MB)	NADES was used for the electropolymerization of PTH-MB polymer films, while ERG increased not only the charge transfer rate but also the surface area of the polymer.	Cyclic voltammetry (CV)	0.51–3.3 mM	0.159 nM	Stability: the sensor current response to 1.0 mM NADH decreased by ca. 10% of the initial current response after 28 days.	[98]
				Amperometry	1.78 μM–0.3 mM	0.13 μM		
C-reactive protein (CRP) as ring-shaped pentameric protein found in blood plasma	Polymerized deep eutectic solvent (PDES)	DNA aptamer immobilized on a graphene nanocomposite functionalized with PDES and coated with gold nanoparticles (AuNPs-PDES-GO)	Covalent functionalization of graphene with PDES boosted its dispersity in several solvents, particularly in aqueous media.	Electrochemical impedance spectroscopy (EIS)	0.001–50 ng mL^−1^	0.0003 ng mL^−1^	Stability: sensor response decreased at 96% of the initial response once operated several times over 10 days. Reproducibility: RSD of 4.6% across five aptasensors.	[101]
Humidity	Betaine/oxalic acid deep eutectic solvent (DES)	Cellulose nanofiber-dispersed graphene (CNF/Gr)	DES was used to extract and esterify the CNF with the aid of ultrasonic treatment.	Resistance	15–99% RH	--	Stability/reproducibility: samples operated under 99% RH for 15 days showed ΔR/R_0_ changes by less than 1%.	[102]
Dopamine	Choline chloride mixed with an oxalic acid anhydride (ChCl:OA)	Regenerated lignin-incorporated cellulose pulp films scribed with CO_2_ laser at 25% of max. laser power for laser-induced graphene (LCPF-LIG-25)	ChCl:OA used for cellulose pretreatment to promote hemicellulose hydrolysis; scribed LIG acted as the working electrode and counter electrode.	Cyclic voltammetry (CV) and differential pulse voltammetry (DPV)	1 μM–40 μM	0.659 μM	--	[103]
Paracetamol (PA)	Choline chloride–urea	Nanocomposite consisting of graphene quantum dots, a deep eutectic solvent, and carboxyl-functionalized multiwall carbon nanotubes (GQDs + DES + MWCNTs-COOH)	Increased anodic peak currents attributed to the high electrical conductivity of the MWCNTs-COOH and DES, and to the high surface area given by the GQDs and the pores generated by the DES.	Differential pulse voltammetry (DPV)	0.030–110 mmol L^−1^	0.010 mmol L^−1^	Stability: peak currents at 96.5% of initial current after 30 h. Repeatability: RSD of 2.7% for six successive determinations of 20.0 mmol L^−1^. Reproducibility: RSDs were 2.90% and 4.09% for intraday and six interday experiments, respectively.	[104]
4-aminophenol (4-AP)					0.050–100 mmol L^−1^	0.017 mmol L^−1^	Stability: peak currents at 96.1% of initial current after 30 h. Repeatability: RSD of 3.1% for six successive determinations of 20.0 mmol L^−1^. Reproducibility: RSDs were 3.22% and 4.17% for intraday and 6 interday experiments, respectively.	
Chloramphenicol	DES prepared from a mixture of ZnCl_2_ and ChCl	Nanocomposite based on covalently functionalizing molecularly imprinted polymers (MIPs) onto the surface of reduced graphene oxide (rGO), which was pretreated with maleic anhydride (MA) via the Diels−Alder reaction in the DES	DES was used as environmentally friendly medium in the Diels−Alder reaction for rGO surface modification in ambient conditions; high electrical conductivity of MIP-functionalized rGO to enhance efficiency as electrochemical sensing materials.	Chronoamperometry, amperometry	0.05–8.0 μM	0.204 μM	Repeatability: testing the same MIP-rGO-based sensor for five subsequent experiments showed a retention at 96.2% of the initial current density response with a relative standard deviation (RSD) of 2.4%. Reproducibility: current responses given by five MIP-rGO-based sensor devices tested for CAP detection, varied with an RSD of 2.35%.	[105]
Oleuropein (OLE)	Natural deep eutectics solvent (NADES)	Graphene oxide (GO) and pencil graphite electrode (PGE) in combination with a buffer modified with a NADES, containing 10% (*v*/*v*) of lactic acid, glucose, and H_2_O (LGH)	NADES enhances electrochemical detection, while PGE is used as low-cost, mechanically stable, carbonaceous electrode material.	Differential pulse voltammetry (DPV)	0.10–37 μM	0.030 μM	Reproducibility: RSD of the oxidation peak current to 18 μM of OLE was 3.16% over five electrodes.	[106]
